# Feasibility and efficacy of a supervised home-based physical exercise program for metastatic cancer patients receiving oral targeted therapy: study protocol for the phase II/III - UNICANCER SdS 01 QUALIOR trial

**DOI:** 10.1186/s12885-020-07381-4

**Published:** 2020-10-09

**Authors:** Florence Joly, Claudia Lefeuvre-Plesse, Claire Garnier-Tixidre, Carole Helissey, Nathalie Menneveau, Alain Zannetti, Sebastien Salas, Nadine Houede, Sophie Abadie-Lacourtoisie, Laetitia Stefani, Soazig Nenan, Isabelle Rieger, Isabelle Durand-Zaleski, Jean-Marc Descotes, Amélie Anota

**Affiliations:** 1 Centre François Baclesse et CHU Côte de Nacre, Caen, France; 2 Centre Eugène Marquis, Rennes, France; 3 Institut Daniel Hollard, Grenoble, France; 4 HIA Begin, Saint Mandé, France; 5 CHRU de Besançon, Besançon, France; 6 CH, Cholet, France; 7 CHU La Timone, Marseille, France; 8 Institut de Cancérologie du Gard, Nîmes, France; 9 ICO René Gauducheau, Nantes, France; 10 CH Annecy Genevois, Pringy, France; 11 Unicancer, Paris, France; 12 Hôpital de l’Hôtel-Dieu, Paris, France; 13 CAMI Sport et Cancer, Neuilly sur seine, France

**Keywords:** Oral targeted therapy, Metastatic cancer, Supervised physical exercise programs, Fatigue, Pain, Quality of life, Psychological and cognitive functions, Adherence to treatment, Supportive care, Medico-economy

## Abstract

**Background:**

Currently, oral targeted therapies are known to be effective and are frequently used to treat metastatic cancer patients, but fatigue is a frequently reported early side effect of these treatments. This fatigue may impact the patient’s treatment adherence and result in a negative impact on quality of life. Physical exercise significantly improved the general well-being and quality of life of advanced cancer patients. However, there is no specific physical activity program adapted for patients with advanced disease.

**Methods:**

QUALIOR is a two-part, randomized, open-label, and multicenter with two arms phase II/III trial. Patients (phase II: *n* = 120; phase III: *n* = 312) with metastatic cancer (breast cancer, kidney cancer, lung cancer, and other cancers [including but not limited to colon cancer, melanoma, sarcoma, or hepatocarcinoma]) treated with a first- or second-line oral targeted therapy without chemotherapy will be included. Patients will be randomized (2:1) to a 3-month supervised home-based standardized physical activity program or to a recommended adapted physical activity (via a booklet). The primary objective of the phase II is to evaluate the feasibility of the supervised program. The primary objective of the phase III is the evaluation of the benefit of the supervised home-based program compare to the recommended program in terms of fatigue and quality of life at 3 months. The secondary objectives aim to evaluate the impact of the supervised program on fatigue over time, pain, physical capacities, psychosocial and cognitive functions, general quality of life, frequency of dose reduction and patients’ adherence to the targeted therapy, overall survival, and progression-free survival. This study will also evaluate the medico-economic impact of supervised program compared to the recommended adapted physical activity program.

**Discussion:**

The aim of this study is to evaluate home-based physical exercise program for metastatic cancer patients treated with oral targeted therapies to help patients to cope with fatigue and improve quality of life.

**Trial registration:**

This trial was registered in ClinicalTrials.gov since May 2017 (NCT03169075).

## Background

Oral targeted therapies (OTT) are effective and frequently used to treat metastatic cancer patients, including metastatic kidney, lung, breast, colon cancer, and melanoma as well as sarcomas [1]. These new-targeted therapies have specific tolerance profiles that differ from those of chemotherapies. Fatigue is one of the major early side effects of OTT. For example, fatigue was reported in 90% of renal cancer patients treated with antiangiogenic tyrosine-kinase inhibitors [2], 60% of breast cancer patients treated with everolimus [3], and in most of the patients that received vemurafenib to treat melanomas [4]. Fatigue results in a decreased quality of life (QoL) and may influence treatment adherence, thus it is important to proposed patients with strategies to prevent and reduce fatigue.

Supervised physical exercise programs (SPEP) followed by patients with localized breast, colon, and prostate cancer have shown an association between an increased overall survival, and significant reduction of fatigue and increased QoL. Several studies revealed that physical exercise significantly improved the general well-being and QoL of advanced cancer patients treated with chemotherapy or hormonal therapy [5]. Only one pilot study was conducted among advanced metastatic cancer patients treated with targeted therapy. This pilot study involving metastatic non-small cell lung cancer patients suggested that physical exercise was feasible and had a positive impact on QoL [6]. For the majority of these studies, the physical activity last for a 3-month period but without standardization of the program [7]. Recent guidelines recommend evaluation and optimization of standardized exercise programs although most of them have been developed for patients with non-metastatic cancer during or after treatments. The majority of these programs were designed for groups or for home-based individual session (with different supports such as video, booklet, or website), but without personalized follow-up. However, for metastatic cancer patients, a category of frail patients, physical activity in groups is not always feasible and the implication of a coach is a major factor for patients’ acceptation of and compliance to a home-based program.

Fatigue reported by patients treated with OTT may be linked with their body mass index (in relation with decrease protein synthesis leading to loss of muscle mass) [8]. Several studies demonstrated a relation between body mass index and survival outcomes in patients treated with targeted therapies for metastatic cancers [9–11]. Furthermore, physical activity may improve patients’ muscle mass, muscular strength, and physical and social components of QoL [12].

The QUALIOR trial is a two parts study which aim to evaluate the feasibility of home-based SPEP assisted with a coach on a 3-month period for patients with metastatic cancer receiving a first- or a second-line OTT (phase II) and the efficacy of this home-based SPEP on patient’s fatigue and QoL (phase III) compared to the recommended program.

## Aim and research questions

The phase II is a randomized non-comparative study to evaluate the feasibility of a home-based, adapted, standardized, and supervised physical exercise program for patients receiving OTT for a metastatic solid tumor (spared in 4 cohorts: breast cancer, kidney cancer, lung cancer, and other cancers [including but not limited to colon cancer, melanoma, sarcoma, or hepatocarcinoma]). The comparative phase III study aims to evaluate the benefit of a SPEP compared to a recommended adapted physical activity, on fatigue and physical dimension of quality of life of such patients at 3 months.

In both phase II and III, the secondary objectives will include the evaluation of the impact of the SPEP on overall and progression-free survival, general quality of life over time, fatigue over time, pain, psychosocial and cognitive functions, muscle mass, muscle density, adipose tissue (visceral and subcutaneous), weight gain, and body mass index. The physical benefit, patients’ observance and tolerance, and the predictive value of the SPEP on survival, general quality of life, and fatigue will also be evaluated. Investigations will also address the toxicity, frequency of dose reduction, and patients’ adherence to the targeted therapy. Finally, this study aims to explore the correlation between fatigue and muscle mass, and between fatigue and muscle density.

The QUALIOR study also encompasses an ancillary evaluation of the medico-economic impact of the SPEP.

## Methods/design

### Study design

**Fig. 1:**
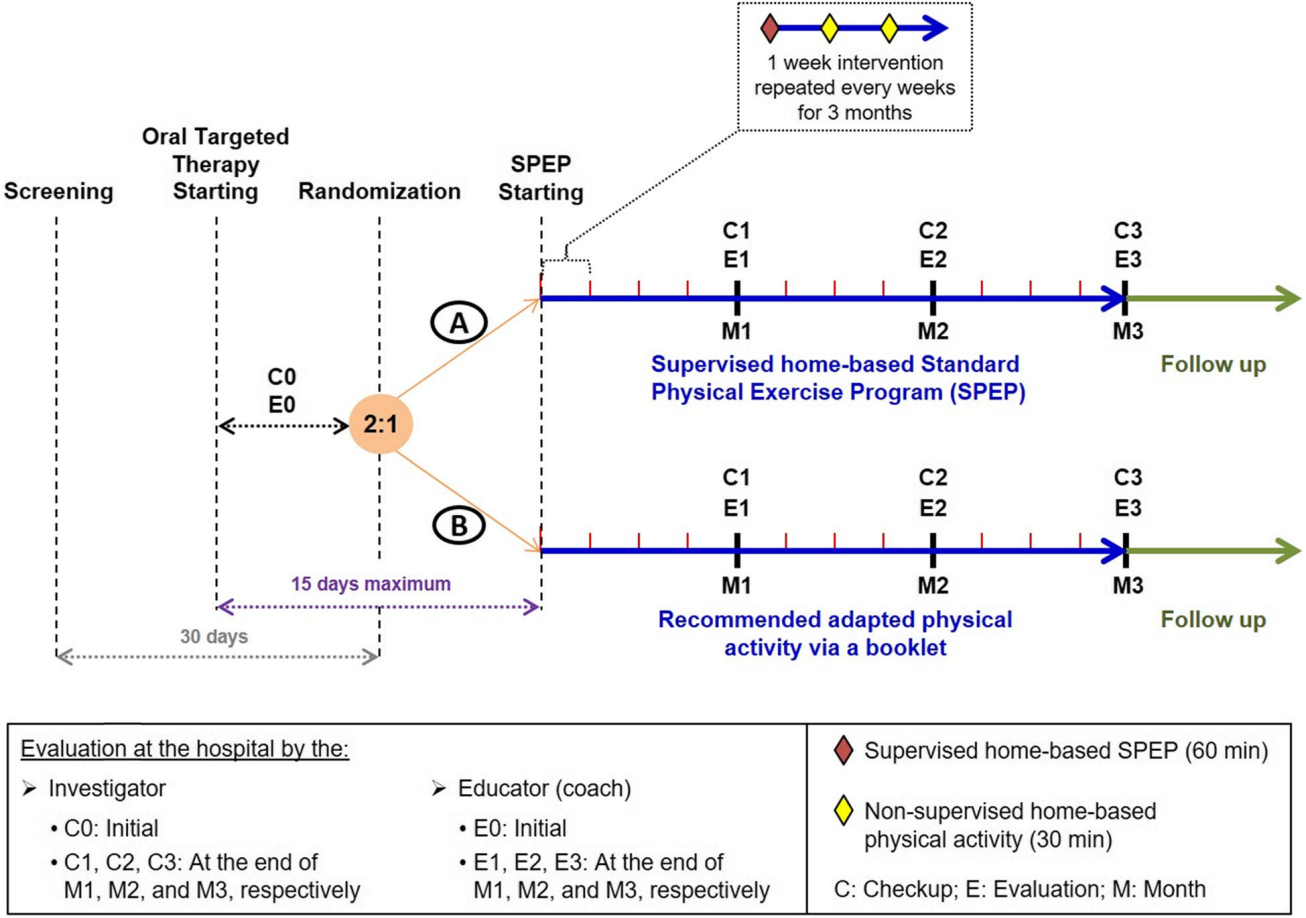
Study design. A baseline assessment will be performed between the initiation of the OTT and the randomization by the clinician (C0) and the educator (E0). Other assessment will be performed monthly by the clinician (C1, C2, C3) and the educator (E1, E2, E3) at the end of month 1 (M1), M2, and M3, respectively. The different assessments will be performed at the hospital

QUALIOR is an open phase II-III, non-blinded, randomized, and multicenter with two arms study (Fig. 1).

### Participant eligibility

#### Inclusion criteria

Eligible patients must be 18 years or older, treated for a metastatic solid tumor, should have received a maximum of two lines of chemotherapy for their metastatic solid tumor, and could have been prescribed a first-line oral targeted therapy. The choice of the oral targeted therapy (that can be associated with hormonal therapy), within the list of drugs with market authorization, is at the investigator discretion. Targeted therapies can include oral anti-angiogenic agents, epithelial growth factor inhibitors, and cyclin-dependent kinase inhibitors. Patients should have a hemoglobin level ≥ 9 g/dL, Eastern Cooperative Oncology Group (ECOG) performance status ≤2, controlled pain, and life expectancy ≥3 months. Patients must be able to comply with the constraints of the standardized supervised physical activity protocol at home and signed informed consent form before study entry. Oral hormonal therapy is not considered as a targeted therapy.

#### Exclusion criteria

comprise patients with known risk of fracture, symptomatic cardiac insufficiency (NYHA-3), respiratory insufficiency (grade 3), intense pain not controlled with analgesic treatment, neuropathy (grade 3), history of cancer in the past 5 years (except basal cell carcinoma adequately treated and in situ cervical cancer treated and cured), or bone metastases with risk of fractures. Patients previously treated with longer than 1 month corticosteroids (dose > 1 mg/kg) before randomization, as well as patient receiving oral targeted therapy with concomitant chemotherapy, or injectable targeted therapy cannot be enrolled in this study. Geographical, sociological, or psychological reasons that could potentially hamper adherence with the study protocol and follow-up schedule, history of non-adherence to medical treatment, reluctance or incapability to conform to the study protocol, and deprivation of liberty or guardianship are also criteria of exclusion.

#### Oral targeted therapies

Investigators will decide which oral targeted therapy best fit their patients’ metastatic disease. The prescribed oral targeted therapies, that must have received marketed authorization, include, but are not limited to the following inhibitors: antiangiogenic tyrosine kinase, epidermal growth factor receptor (EGFR), cyclin-dependent kinases, BRAF, ALK, mTOR, and PARP.

### Interventions

#### Experimental arm (SPEP): program A

All participants in the experimental arm will have to start the SPEP session within 15 days following the initiation of the oral targeted therapy.

This program will last 3 months with a total of 12 SPEP sessions (one weekly) plus 24 non-supervised exercise sessions (twice a week). An expert coordinator coach has elaborated three levels of difficulties for this program.

The home-based SPEP sessions are composed of two dimensions: development of the musculoskeletal strength and patients’ endurance (muscular reinforcement of the cardiorespiratory system). These sessions will be supervised by a coach (trained physical educator or physiotherapist) specifically trained for this study, with respect to the pathology and the clinical study. Before each SPEP session, using a visual analogic scale, the coach will determine the patient’s level of fatigue to adjust the intensity, the repetition, and the speed of execution of the exercises proposed during the 60-min session. The coach will report the information of duration and intensity of the sessions in a standardized document.

Between each SPEP session, patients will follow two non-supervised exercise sessions (planned with the coach) consisting of a minimum of 30 min and maximum of 60 min of walk, running, or biking, depending on the patient’s level of fatigue (estimated by patients themselves). For these non-supervised sessions, the coach will provide patient with a heart rate monitor (connected watch) and a dedicated booklet containing personalized physical activity recommendations.

#### Control arm (recommended adapted physical activity): program B

Patients randomized in the control arm will receive a booklet containing general physical activity recommendations to reduce the fatigue resulting from cancer treatments (including some proposition of exercises): information and advice to adapt daily routine, preserve valuable time with family and friends, and maintain physical activity to ensure well-being without exceeding their limits. Patients could choose the exercises to follow within the booklet. Each session proposed in the booklet will last 30 min minimum.

### Study outcomes

#### Primary outcome

##### ***Feasibility****,* phase II non-comparative study

The feasibility will be assessed using the rate of patients that participated adequately in the SPEP. The SPEP participation will be considered sufficient if the patient complete at least 50% of the theoretically planned sessions in the 3 months (supervised and non-supervised).

##### ***Efficacy****,* phase III comparative study

The co-primary endpoint is the fatigue score (using FACT-F questionnaire) and the physical well-being score (using FACT-G questionnaire) evaluated after 3 months. A difference of at least 2.5 points for FACT-F and at least 5 points for the physical well-being score of FACT-G will be considered clinically significant. SPEP superiority over the recommended adapted physical activity program will be demonstrated if at least one of the targeted dimensions (FACT-F or the physical well-being of the FACT-G) is significantly improved without deterioration of the other dimension.

#### Secondary outcomes

Progression free survival is defined as the time from randomization to date of disease progression or death of any cause, whichever occurs first.

Overall survival is defined as the time from the date of randomization to date death of any cause.

Toxicity of the targeted therapy will be assessed using the NCI-CTCAE v4.03. Data concerning the class, frequency, and severity of adverse events will be collected and analyzed. The data will be collected at baseline, monthly during the 3-month program, then every 3 months during the first year of follow-up.

Adherence to targeted therapy will be evaluated by the Morisky-Green questionnaire at baseline then monthly during the 3-month program. This questionnaire, composed of six items related to patient’s adherence to the scheduled treatment, is regularly used to evaluate adherence to oral therapies. Scoring is performed through sum of item scores. Higher scores indicate better adherence to treatment.

For the outcomes described below, patients will fill the French versions of the specific questionnaires at baseline, monthly during the 3-month program, then every 3 months during the first year of follow-up, otherwise indicated. For each questionnaire but EQ-5D-3L, scoring is performed through a simple sum of item scores using the scoring recommendations of the authors.

Quality of life (QoL) will be assessed using the FACT-G and the 3-level version of EuroQol-5D (EQ-5D-3L) questionnaires. FACT-G is composed of 27 items related to patient’s general well-being grouped into four well-being subscales: physical, social/family, emotional, and functional. One score is generated for each subscale and a global QoL score is generated from all items corresponding to the total FACT-G score. For each dimension, a higher score represents a better QoL level. EQ-5D-3L consists of a patient self-rate health on a visual analogue scale (VAS) plus a descriptive system of five dimensions: mobility, self-care, usual activities, pain/discomfort, and anxiety/depression. Each dimension has three levels of perceived problems. Patient decision results into a 1-digit number for each dimension that can be combined into a 5-digit number that describes the patient’s health state.

Fatigue will be assessed using the functional assessment of cancer therapy-fatigue (FACT-F; 13 items related to patient’s fatigue) questionnaire and the VAS (fatigue; 0–10 scale). For the FACT-F questionnaire, a higher score represent a lower level of fatigue while for the VAS, a higher score represent a higher level of fatigue. For the experimental arm, in addition to the above mentioned schedule, the coach will systematically evaluate the patient’s fatigue by VAS before and after each home-based supervised session.

Pain will be evaluated by VAS (pain; 0–10 scale), a higher score represent a higher level of pain. For the experimental arm, in addition to the above mentioned schedule, the coach will systematically evaluate the patient’s pain by VAS before and after each home-based supervised session.

The physical benefit will be assessed using a rating grid and the short version of the international physical activity questionnaire (IPAQ). The rating grid take into account the physical activity progression plus the physical performance: the 6-min walking test, muscle strength using a dynamometer, muscle function (resistance, flexibility, and stability), and biometry (body mass index, muscle mass index, and amount of body fat). To evaluate patients, the coach will use this grid at baseline then monthly during the 3-month program. The short version of the IPAQ, which comprises four generic items to collect data on patient’s health–related physical activity, will be completed at baseline, month 3 of the program (M3), then every 3 months during the first year of follow-up.

Psychological and cognitive functions will be evaluated using the hospital anxiety and depression scale (HADS) and the functional assessment of cancer therapy–cognitive function (FACT-Cog) version 3 questionnaires. The HADS is a 14 items questionnaire: 7 items related to anxiety and 7 items related to depression scored on a scale. Scores for each subscale (anxiety and depression) range from 0 to 21 and the entire scale (emotional distress) range from 0 to 42, with higher scores indicating more distress. The FACT-Cog is a self-assessment questionnaire to estimate memory, attention, concentration, language, and thinking abilities. This questionnaire, composed of 37 items consists of four subscales: cognitive impairments perceived by the patient (20 items), comments from others (4 items), cognitive abilities perceived by the patient (9 items), and impact on quality of life (4 items). Higher scores indicate greater impairment of cognitive functions.

Body composition will be evaluated by the body mass index and imaging technique described by Martin et al. [13]. Imaging data of visceral adipose tissue, sub-cutaneous adipose tissue, skeletal muscle mass, and skeletal muscle density obtained by computed tomography of thorax, abdomen and pelvis (CT-TAP) scan at baseline, 3 months, and 6 months will be centrally reviewed. Since normal values for skeletal muscle mass, adipose tissues, and threshold values determining sarcopenia are gender-dependent, results from men and women will be analyzed separately.

Anorexia and food intake will be evaluated using the anorexia/cachexia subscale (A/CS) of the functional assessment of anorexia/cachexia therapy (FAACT) questionnaire. The 12 items of the FAACT-A/CS are scored on a 5-point Likert scale. A lower score indicates less appetite. The VAS for appetite will also be performed at baseline and at M3. A state of anorexia will be validated if the FAACT-A/CS score is ≤37 or if the VAS ≤7 [14].

#### Ancillary outcome

Medico-economic impact of the program for centers and patients. The cost effectiveness of the program will be assessed by comparing the full costs of implementing and operating the SPEP in the intervention centers to the costs in the control group, and relating the cost difference to the difference in health outcomes. The cost for the healthcare system will be estimated from the total cancer-related costs over the follow-up period (primary time horizon = 1 year, secondary time horizon = 5 years). The analysis will follow the French Health Authority and the Consolidated Health Economic Evaluation Reporting Standards (CHEERS) guidelines on economic evaluation in health care [15, 16]. Hospital costs will be calculated as the diagnosis related group-specific costs weighted by the actual patients’ length of stay. Data collection for out of hospital resources will focus on targeted therapies that represent the highest cost item. The economic evaluation of SPEP compared to adapted physical activity will be expressed as the incremental cost-effectiveness ratio in euro (€) per quality-adjusted life year. We will present the costs differences from the perspective of the health care in the primary analysis and from the perspective of the patients in the secondary analysis. The 95% confidence interval will be estimated by bootstrap method. We will compare the incremental cost-effectiveness ratio to the usually applied thresholds of €50,000–100,000 per quality-adjusted life year and calculate the probability of cost-effectiveness from the bootstrapped probabilistic sensitivity analysis.

### Study visits

Patients will be monitored from the date of their randomization until the date of death, withdrawal of consent, or loss to follow-up. The investigators will perform checkup (C) while the coaches will perform evaluation (E). Before any physical exercise, the coach has to monitor patient’s resting pulse rate, oxygen saturation, and blood pressure. Table 1 details the examinations and visits schedule.

**Table 1:** Examinations and visits schedule

	Baseline	Physical activity program period			Follow-up 1 (1st year)	Follow-up 2 (5 last years)^a^
		M1	M2	M3		
Inform Consent form	●					
**CLINICAL EXAMINATION**						
Observance of oral targeted therapy^b^	●	●	●	●	●^c^	
Medical history and comorbidities	●					
Toxicities related to targeted therapy^d^	●	●	●	●	Every 3 months	
Adverse events related to physical activity^d^		●	●	●		
Clinical examination & vital signs^e^	●	●	●	●	Every 3 months	Every 3 months^k^
Survival Status		●	●	●	Every 3 months	Every 3 months^k^
**QUESTIONAIRES**						
General QoL (FACT-G, EQ-5D-3L)^f^	●	●	●	●	Every 3 months	
Fatigue (FACT-F)^f^	●	●	●	●	Every 3 months	
Nutrition (FAACT module AC/S)	●	●	●	●	Every 3 months	
Cognitive Function (FACT-Cog)	●	●	●	●	Every 3 months	
Food intake VAS	●			●		
Pain and fatigue VAS	●	●^g^	●^g^	●^g^	Every 3 months	
Adherence to targeted therapy (Morisky-Green)	●	●	●	●		
Anxiety and Depression (HADS)	●	●	●	●		
Daily physical activity						
○ IPAQ	●			●	Every 3 months	
○ Booklet		●	●	●		
○ Questionning about the continuation of physical activity					Every 3 months	
**OTHER ASSESSMENTS**						
Muscle mass, muscle density, adipose tissue (VAT and SCAT), Evaluation (TAP-CT)	●			●	●^h^	
Physical capacities^i^	E0	E1	E2	E3		
**PARACLINICAL EXAMINATIONS**						
Tumor evaluation^j^	●			●	●^l^	●^k, l^
Scintigraphy	●			●	●	
MRI	●			●	●	
**BIOLOGICAL TEST**						
Biological Assessment^k^	●	●	●	●		
**INTERVENTION**						
**Experimental Arm** SPEP Program		Weekly: - 1 supervised SPEP session at the patient’s home - 2 non-supervised sessions				
**TRANSLATIONAL RESEARCH**						
Blood samples^m^	●			●		

*A/CS* Anorexia/cachexia subscale, *ALAT* Alanine transaminase, *ASAT* Aspartate transaminase, *ALP* Alkaline phosphatase, *aPTT* Partial thromboplastin time, *Ca* Calcium, *CBC* Complete blood count, *CRP* C-reactive protein, *E* Evaluation, *EQ-5D-3L* EuroQol-5 dimensions-3 levels, *FAACT* Functional assessment of anorexia/cachexia therapy, *FACT-Cog* Functional assessment of cancer therapy-cognitive, *FACT-F* Functional assessment of cancer therapy-fatigue, *FACT-G* Functional assessment of cancer therapy-general, *γGT* Gamma glutamyl transpeptidase, *HADS* Hospital anxiety and depression scale, *HDL* High-density lipoprotein, *IPAQ* International physical activity questionnaire, *K* Potassium, *LDL* low-density lipoprotein, *M* Month, *Na* Sodium, *NCI-CTCAE* National cancer institute - common terminology criteria for adverse events, *PT/INR* Prothrombin time and international normalized ratio, *PWB* Physical Well Being, *QoL* Quality of life, *SCAT* Sub-cutaneous adipose tissue, *SPEP* Supervised physical exercise program, *T4* Thyroxine, *TAP-CT* Computed tomography of thorax, abdomen and pelvis, *TP* Prothrombin, *TSH* thyroid-stimulating hormone, *VAS* Visual analogue scale, *VAT* Visceral adipose tissue

^a^Every 3 months until disease progression (Tumor assessment according to standard procedures in centers). After progression, the survival status will be evaluated every 3 months until the end of the follow-up period or death

^b^Using the Morisky-Green questionnaire

^c^Until disease progression, death, toxicities, or patient/ investigator decision

^d^Using the NCI-CTCAE V4.03

^e^Pulse rate, blood pressure, temperature, weight

^f^The co-primary endpoint criteria are the fatigue scores (FACT-F) and the physical dimension scores (FACT-G – PWB) evaluated after 3 months

^g^Every week before and after SPEP for Arm A, once a week for Arm B

^h^Scanner at baseline, at 3 months (during the physical activity phase), and at the first follow-up visit (at 6 months after initiation of the physical activity for the SPEP group and 6 months after randomization for the standard group)

^i^The 6-min walking test, muscle force and function, pulse rate, height, weight, body surface area

^j^Tomodensitometry

^k^Hematology (CBC and platelet count), coagulation profile (PT/INR and aPTT), ionogram (NA, K, and Ca), lipid profile (cholesterol total, HDL, HDL, and triglycerides), kidney functions (bilirubin [total, direct, and indirect], ALAT, ASAT, γGT, and ALP), kidney functions (creatinine, creatinine clearance), albumin, thyroid (TSH, T4), CRP

^l^According to standard of care in the center, with an evaluation during the first visit (6 months ±15 days from SPEP initiation [Arm A] or randomization [Arm B])

^m^C-Peptide, Insulin Growth Factor 1, insulin, estradiol, and leptin

#### Inclusion (baseline) visit

Eligible patients with signed informed consent form will have baseline visit at the hospital. Baseline checkup and evaluation will be performed the same day before patient randomization.

Checkup (C0):

- Clinical examinations
- Paraclinical examinations
- Biological tests
- Questionnaires: VAS (fatigue, pain, and food intake), FACT-G, FACT-F, FACT-Cog, EQ-5D-3L, FAACT-AC/S, and HADS
- Blood samples for translational research (if consented)

Evaluation (E0):

- Physical capacities
- Questionnaires: VAS (fatigue and pain) and IPAQ

#### Interventional visits

During the intervention phase, the investigator and coach will perform the following checkups and evaluations:

- ➢ C1 and E1: 1 month ±3 days from SPEP initiation [experimental arm] or randomization [control arm]
- ➢ C2 and E2: 2 months ±3 days from SPEP initiation [experimental arm] or randomization [control arm]

Monthly checkup and evaluation will be performed during the same day for each visit.

Monthly checkup (C1, C2): At the hospital, for all patients.

- Clinical examinations
- Biological tests
- Questionnaires: VAS (fatigue and pain), FACT-G, FACT-F, FACT-Cog, EQ-5D-3L, FAACT-AC/S, and HADS

Weekly evaluations: At home, for patients in the experimental arm only.

- Resting pulse rate
- Questionnaires: VAS (fatigue and pain)

Monthly evaluations (E1, E2): At the hospital, for all patients.

- Physical capacities
- Questionnaires: VAS (fatigue and pain)

#### End of study visit

Checkup (C3):

- Clinical examinations
- Paraclinical examinations
- Biological tests
- Questionnaires: VAS (Fatigue, pain, and food intake), FACT-G, FACT-F, FACT-Cog, EQ-5D-3L, FAACT-AC/S, and HADS
- Blood samples for translational research (if consented)

Evaluation (E3):

- Physical capacities
- Questionnaires: VAS (fatigue and pain) and IPAQ

#### Follow-up visits

The investigator will follow patients for a maximum of 6 years after the end of the program.

Follow-up 1 (1st year):

- Clinical examinations (every 3 months)
- Paraclinical examinations
- Questionnaires (every 3 months): VAS (fatigue and pain), FACT-G, FACT-F, FACT-Cog, EQ-5D-3L, FAACT-AC/S, and IPAQ
- Blood samples for translational research (if consented)

Follow-up 2 (last 5 years):

- Before disease progression (every 3 months)
  - ❖ Biological tests
  - ❖ Clinical and vital signs
  - ❖ Survival status
  - ❖ Tumor evaluation
- After progression (every 3 months)
  - ❖ Survival status until death or end of follow-up.

### Statistical plan

#### Sample size

The primary objective for the phase II is the feasibility of the study. The SPEP participation will be considered sufficient if a patient has performed at least 50% of the theoretically planned sessions within 3 months (supervised and non-supervised). With a unilateral alpha risk of 5%, a statistical power of 85%, a probability of inefficiency maximum of 50%, a probability of minimum efficiency of 65%, and a 5% lost to follow-up rate, 120 patients will need to be randomized in the phase II with a 2:1 ratio (74 patients in the SPEP; 37 patients in the control arm of recommended adapted physical activity). Patients will be included in a non-competitive manner in the different cohorts. An interim analysis is planned when 37 patients will be randomized in the SPEP arm with rejection of H0 if at least 27 patients have completed at least 50% of the planned sessions; rejection of H1 (futility) if 19 patients or less have completed at least 50% of the planned sessions.

The phase III study will evaluate the 3-month efficacy of the SPEP, compared to recommended adapted physical activity. The 120 patients randomized in the phase II will be included in the phase III analysis. The co-primary endpoint criteria are the fatigue score (using FACT-F) and the physical well-being score (using FACT-G) evaluated after 3 months. SPEP superiority over the recommended adapted physical activity program will be demonstrated if at least one of the criteria (FACT-F or FACT-G) increase significantly without deterioration of other criteria. To demonstrate a clinically significant difference of at least 5 points (standard deviation: 13) for the physical well-being dimension of the FACT-G questionnaire and at least 2.5 points (standard deviation: 6.5) for FACT-F questionnaire at 3 months [17, 18], with a bilateral alpha risk of 2.5%, a statistical power of 80%, and a 5% lost to follow-up rate, 312 patients will need to be randomized in the phase III. The inclusions will be non-competitive between the 4 cohorts (78 patients per cohort). A 30-month inclusion period is predicted for the recruitment of these 312 patients in 18 French centers. An interim analysis is planned at the end of the phase III corresponding to 39.5% of patients randomized in order to reject H0 or H1 according to alpha spending function.

### Statistical analysis

For the phase II, all the analyses will be non-comparative and performed on the intention-to-treat (ITT) population: all patients included in the study irrespective of the respect of the study.

For the phase III, analyses will be performed on the modified ITT (mITT) population: ITT patients with FACT-F and physical well-being score available at baseline and M3. Longitudinal analysis of QoL data will be performed on a mITT2 population: ITT patients with FACT-F and physical well-being score available at baseline. Quantitative analyses will be described by the mean (standard deviation) and median (minimum-maximum) values while qualitative and categorical analyses will be reported by frequency and percentage. A description of FACT-F, FACT-G, and FACT-Cog scores will be done by arm for each time point with the mean (standard deviation) and median (minimum-maximum) values. The statistical analysis will be performed using t-test, log-rank, stratified log-rank, invariable analysis, and multivariable analysis.

For the co-primary endpoint, a t-test will be done to compare FACT-F and physical well-being scores at 3 months according to the study arms, at the statistical level of 2.5%, after checking the normality of the scores and potential transformation of the scores. An analysis of variance for repeated measure will also be done taking into account the baseline score. Then, some univariate and multivariate models will be done in order to identify factors associated with the change of fatigue and physical well-being score between baseline and 3 months. All factors with an univariate *p*-value < 0.1 will be eligible for the multivariate models.

### Randomization

Randomization, achieved using the Clinsight® software, will take place within 15 days of the initiation of the targeted therapy. Patients will be allocated to either the experimental SPEP group or the recommended adapted physical activity group in a ratio 2:1. Randomization will be stratified per site, baseline level of fatigue, targeted therapy, number of lines of prior therapy in the metastatic status, and age.

## Discussion

One of the most significant advance in the treatment of cancer in the past few decades has been the introduction of targeted therapies. Compare to chemotherapeutic drugs, OTT are more convenient (with some oral routes) and precise. However, OTT induce specific side effects, and fatigue is one of the most important early side effect reported by patients. This fatigue causes significant impairment in QoL and may also be a predictor of shorter survival in cancer patients [12]. Some reports suggested that fatigue related to cancer treatments differs from, and is more debilitation than normal fatigue induced by other causes such as sleep disturbance and exercise. Furthermore, patients described fatigue related to cancer treatments as unusual, excessive, unrelated to activity, and not relieved by sleep or rest [19]. Cancer-related fatigue is multi-dimensional and may have physical, mental, and emotional manifestations including generalized weakness, diminished concentration or attention, decreased motivation or interest to engage in usual activities, and emotional lability [20].

This present article describes the QUALIOR study protocol of a randomized controlled trial of a home-based SPEP for metastatic cancer patients treated with oral targeted therapies. The originality of our study lies on the presence of a personal coach for a supervised home-based and well-designed standardized physical exercise program for the management of advanced cancer patients treated with OTT. The first part of the study (phase II) is designed to evaluate the feasibility of the home-based SPEP in this group of metastatic cancer patients who are particularly frail. The second part of the study (phase III) aims to demonstrate that supervised physical exercise may help these patients to minimize the OTT-related fatigue and improve their QoL. We expect that the SPEP will prove a positive impact on patient fatigue and quality of life compared to the recommended adapted physical activity. Secondary endpoints will explore if SPEP may improve patient survival along with a decrease of OTT toxicity. Most of the parameters studied in this trial will be evaluated using approved French versions of commonly used self-assessment questionnaires.

Cancer treatment-related fatigue may lead to poor adherence to treatment and is often a reason for patients’ treatment discontinuation. Cancer treatment adherence is crucial to obtain optimal health outcomes. Cancer treatment non-adherence leads to high treatment failure and decreased survival [21]. The design of the QUALIOR study will allow to investigate if the SPEP-decreased OTT-related fatigue may improve OTT adherence, and increase patient survival (progression-free and overall survival).

Apart from the fact that treatment-related fatigue causes significant impairment in overall quality of life during treatment, has a negative impact on social relationships, mood, daily activities, and patient survival [22], it also has negative influence on work abilities and by consequence an important economic implications. These economic impacts not only involve patients but also caregivers who often need to reduce work hours, accept fewer responsibilities, take days off, or ultimately stop working [23]. Furthermore, treatment-related fatigue has a cost for the society and the health care system [21]. For these reason, an ancillary research of the QUALIOR study will assess the economic impact of the SPEP compare to the recommended adapted physical activity.

In conclusion, the QUALIOR study aim to answer whether or not supervised physical exercise program at home is feasible among a group of metastatic cancer patients treated with oral targeted therapy and may decrease fatigue, the major side effect observed for these patients, resulting in an improved drug efficacy and patients’ quality of life.

## Trial status

Recruitment began 11 July 2017 and is still ongoing at the date of publication.

## Data Availability

Not applicable

## Abbreviations

A/CS: Anorexia/cachexia subscale
ALAT: Alanine transaminase
ASAT: Aspartate transaminase
ALP: Alkaline phosphatase
aPTT: Partial thromboplastin time
C: Checkup
Ca: Calcium
CBC: Complete blood count
E: Evaluation
ECOG: Eastern Cooperative Oncology Group
EGFR: Epidermal growth factor receptor
EQ-5D-3L: EuroQol-5 dimensions-3 levels
FAACT: Functional assessment of anorexia/cachexia therapy
FACT-Cog: Functional assessment of cancer therapy-cognitive
FACT-F: Functional assessment of cancer therapy-fatigue
FACT-G: Functional assessment of cancer therapy-general
γGT: Gamma glutamyl transpeptidase
HADS: Hospital anxiety and depression scale
HDL: High-density lipoprotein
IPAQ: International physical activity questionnaire
ITT: Intention-to-treat
K: Potassium
LDL: Low-density lipoprotein
M: Month
mITT: modified intention-to-treat
MRI: Magnetic resonance imaging
Na: Sodium
NCI-CTCAE: National cancer institute - common terminology criteria for adverse events
OTT: Oral targeted therapy
PARP: Poly(ADP-ribose) polymerase
PT/INR: Prothrombin time and international normalized ratio
QoL: Quality of life
SCAT: Sub-cutaneous adipose tissue
SPEP: Supervised physical exercise program
T4: Thyroxine
TAP-CT: Computed tomography of thorax, abdomen and pelvis
TP: Prothrombin
TSH: Thyroid-stimulating hormone
VAS: Visual analogue scale
VAT: Visceral adipose tissue
